# Supplementary material for: vcfdist: accurately benchmarking phased small variant calls in human genomes
Source: Nat Commun. 2023 Dec 9;14:8149. doi: 10.1038/s41467-023-43876-x (PMC10710436; doi:10.1038/s41467-023-43876-x)
Supplement: Supplementary file 1 — Supplementary Information [file 41467_2023_43876_MOESM1_ESM.pdf]

vcfdist: Accurately benchmarking phased small variant calls in human genomes

Supplementary Information

Reference `AAGGAAATC` **Query** `ATCGAAAATC`

Alignment

(a)

```
AAGGAAA-TC
.  .... ..
ATCGAAAATC
```

VCF

POS	REF	ALT
2	AG	TC
6	AATC	AAATC

Original

(b)

```
AAGGAAA-TC
.  .... ..
ATCGAAAATC
```

POS	REF	ALT
2	A	T
3	G	C
6	AATC	AAATC

Decomposed

(c)

```
AAGGAAA-TC
.  .... ..
ATCGAAAATC
```

POS	REF	ALT
2	A	T
3	G	C
7	A	AA

Trimmed

(d)

```
AAGG-AAATC
.  . .....
ATCGAAAATC
```

POS	REF	ALT
2	A	T
3	G	C
4	G	GA

Left shifted

Supplementary Figure 1: **Variant normalization example.** Variant normalization of a multi-nucleotide polymorphism (MNP) and single base insertion. In **(a)**, the original MNP and insertion is reported. In **(b)**, the MNP is decomposed into two single nucleotide polymorphisms (SNPs). In **(c)**, the unchanged reference bases reported in the VCF on either side of the insertion are trimmed. In **(d)**, the insertion is left-shifted as far as possible.

(a)

Reference `TTCCTTTCTTTCTTCCTTTCTTTCTTTCTT`

Query `TTCCTTTCTTCTTTCTT` **Truth** `TTCCTTTCCTTCTT`

Query VCF

POS	REF	ALT
8	CTTTCTTCCTTTCT	C

Alt. Query VCF

POS	REF	ALT
3	CCTTTCTTTCTTCCTTT	C
24	C	CTTC

Truth VCF

POS	REF	ALT
3	CCTTTCTTTCTTCCTTT	C
25	T	C

(b)

vcfeval INDEL Summary Statistics

	TP	FP	FN	PP	Prec.	Recall	F1	F1 Q-score
Query	0	1	1	0	0.00	0.00	0.00	0.00
Alt. Query	1	1	0	0	0.50	1.00	0.67	4.77

(c)

vcfdist INDEL Summary Statistics

	TP	FP	FN	PP	Prec.	Recall	F1	F1 Q-score
Query	0	0	0	1	0.82	0.82	0.82	7.53
Alt. Query	1	1	0	0	0.50	1.00	0.67	4.77

(d)

vcfdist Distance Summary

	ED	DE	DE Q-score	ED Q-score	ALN Q-Score
Reference	17	2			
Query	3	2	7.27	0.00	4.56
Alt. Query	3	2	7.27	0.00	4.56

Supplementary Figure 2: **A real-world vcfdist partial credit example.** Example vcfeval and vcfdist evaluation of Truth Challenge V2 winning submission `K4GT3` on GRCh38 region `chr5:140,941,200-140,941,230`. This region is within the protocadherin alpha gene cluster, genes *PCDHA1-PCDHA13* in the CMRG dataset. **(a)** Reference, query, and truth sequences, as well as the query and truth VCFs. An alternate query VCF representation (resulting in the same query sequence) is shown as well. **(b)** vcfeval and **(c)** vcfdist count of true positive, false positive, false negative, and partial positive INDEL variants, as well as the calculated precision, recall, and F1 scores. Note that although both the original and alternate query VCF variant calls result in the exact same query sequence, the summary statistics differ. **(d)** Distance-based summary statistics reported by vcfdist (edit distance and distinct edits), which are independent of variant representation. An explanation of these summary statistics can be found in the *Methods* section.

(a)

**Tools**	**Configs**	m	x	o_1	e_1	o_2	e_2
BWA, GRAF, DRAGEN	default	-1	4	6	1		
minimap2	`sr`	-2	8	12	2	32	1
winnowmap, minimap2	default, `map-ont`	-2	4	4	2	24	1
minimap2	`map-pb`	-1	4	6	2	26	1
pbmm2	default, `CCS`	-2	5	5	4	56	2
minimap2	`asm5`	-1	19	39	3	81	1
minimap2	`asm10`	-1	9	16	2	41	1
ngmlr	`pacbio`	2	-5	-5	-5		-1
ngmlr	`ont`	3	-3	-1	-1		-0.5

(b)

**Tool**	**Config**	m	x	o_1	e_1	o_2	e_2
BWA, GRAF, DRAGEN	default	0	1	1.3	0.3		
minimap2	`sr`	0	1	1.3	0.3	2.5	0.2
winnowmap, minimap2	default, `map-ont`	0	1	0.833	0.5	4.167	0.333
minimap2	`map-pb`	0	1	1.3	0.5	5.3	0.3
pbmm2	default, `CCS`	0	1	0.857	0.714	8.14	0.429
minimap2	`asm5`	0	1	1.975	0.175	4.075	0.075
minimap2	`asm10`	0	1	1.65	0.25	4.15	0.15
ngmlr	`pacbio`	0	1	0	0.917		0.25
ngmlr	`ont`	0	1	0	0.417		0.333

Supplementary Figure 3: **Affine gap parameters from the literature.** **(a)** Original and **(b)** normalized affine-gap parameters for tools used in the pFDA Truth Challenge V2. Normalized parameters are plotted in Figure 2.

(a)

TYPE	THRESH	TRUTH TP	QUERY TP	TRUTH FN	QUERY FP	PREC	RECALL	F1 SCORE	F1 QSCORE
SNP	Q >= 0	4636790	4640475	5502	32780	0.993	0.999	0.996	23.78
SNP	Q >= 0	4636790	4640475	5502	32780	0.993	0.999	0.996	23.78
INDEL	Q >= 0	744931	742121	3600	5990	0.991	0.994	0.993	21.27
INDEL	Q >= 7	744844	742055	3687	5776	0.992	0.994	0.993	21.31

(b)

TYPE	THRESH	EDIT DIST	DISTINCT EDITS	ED QSCORE	DE QSCORE	ALN QSCORE
ALL	Q >= 0	62704	48117	20.36	20.50	20.44
ALL	Q >= 6	62612	48126	20.37	20.50	20.45
ALL	Q >= 61	6819150	5394057	0.00	0.00	0.00
SNP	Q >= 0	37545	37545	20.92	20.92	
SNP	Q >= 0	37545	37545	20.92	20.92	
SNP	Q >= 61	4643522	4643522	0.00	0.00	
INDEL	Q >= 0	25159	10572	19.37	18.51	
INDEL	Q >= 8	24857	10488	19.42	18.54	
INDEL	Q >= 61	2175628	750535	0.00	0.00	

Supplementary Figure 4: **Example vcfdist output.** Example vcfdist output, evaluating pFDA Truth Challenge V2 winner `K4GT3`. **(a)** Precision, recall, and F-score summary statistics **(b)** Distinct edits and edit distance summary statistics. For each type, results are shown for when no variants are filtered (`Q >= 0`), at the optimal Q-score cutoff, and in (b) when all variants are filtered (`Q >= 61`).

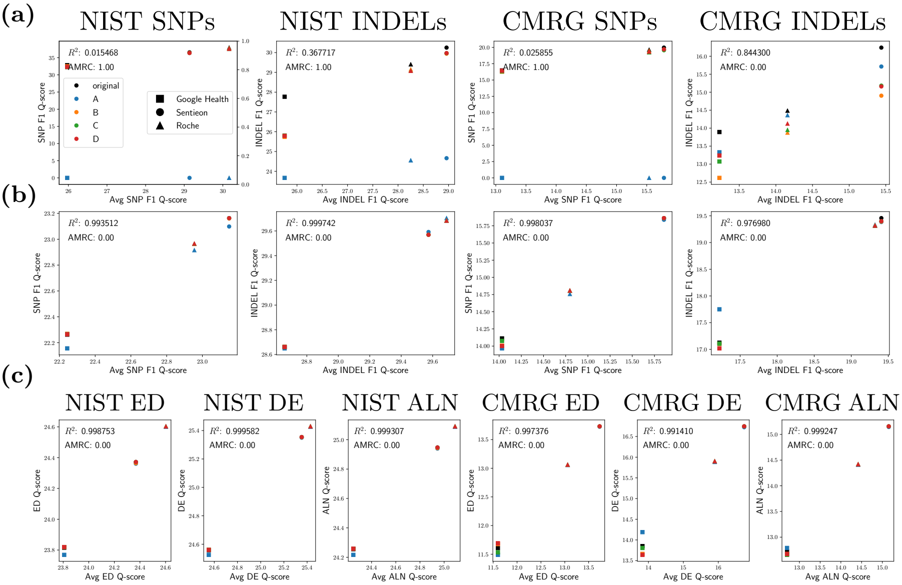

Supplementary Figure 5: **Evaluation stability results for vcfeval and vcfdist top performing submissions.** **(a)** vcfeval and **(b)** vcfdist F1 Q-score plots for the three overall winning Truth Challenge V2 submissions using multiple sequencing technologies on both the NIST and CMRG datasets. **(c)** The same information, using new sequence distance-based summary metrics (see *Methods* for a full explanation of each). On each graph, average Q-score is plotted against the Q-score for the original representation and at points A, B, C, and D (see Figure 2). Source data are provided as a Source Data file.

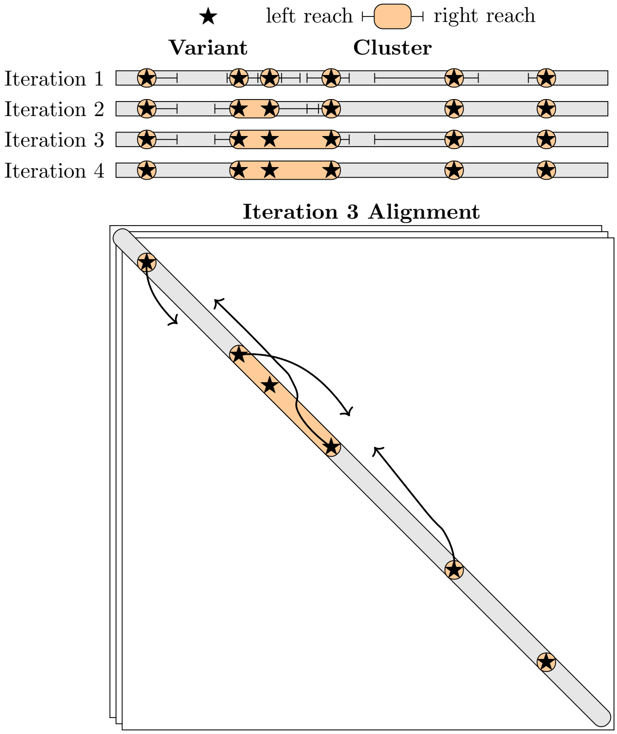

Supplementary Figure 6: **Clustering algorithm overview.** Clusters are initialized to each variant and then iteratively grown by extending leftwards (from each active cluster's end) and rightwards (from each active cluster's start) under Smith-Waterman alignment (disallowing reference diagonal matches, the gray shaded region) until a score limit is reached. Clusters are considered "active" if adjacent to a cluster that was merged in a previous iteration. Two clusters are considered dependent if the leftmost extension of the right cluster overlaps with the rightmost extension of the left cluster. Dependent clusters are merged after each iteration until no more clusters are active.

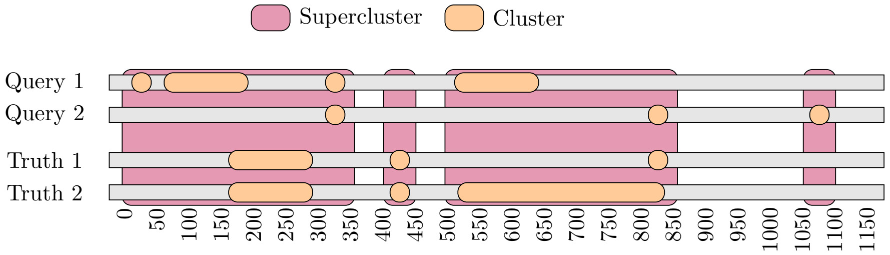

Supplementary Figure 7: **Superclustering algorithm overview.** An example demonstrating how variant clusters are grouped into superclusters, which span all haplotypes. Clusters within 50 bases of another cluster (on any haplotype) are merged into a single supercluster.

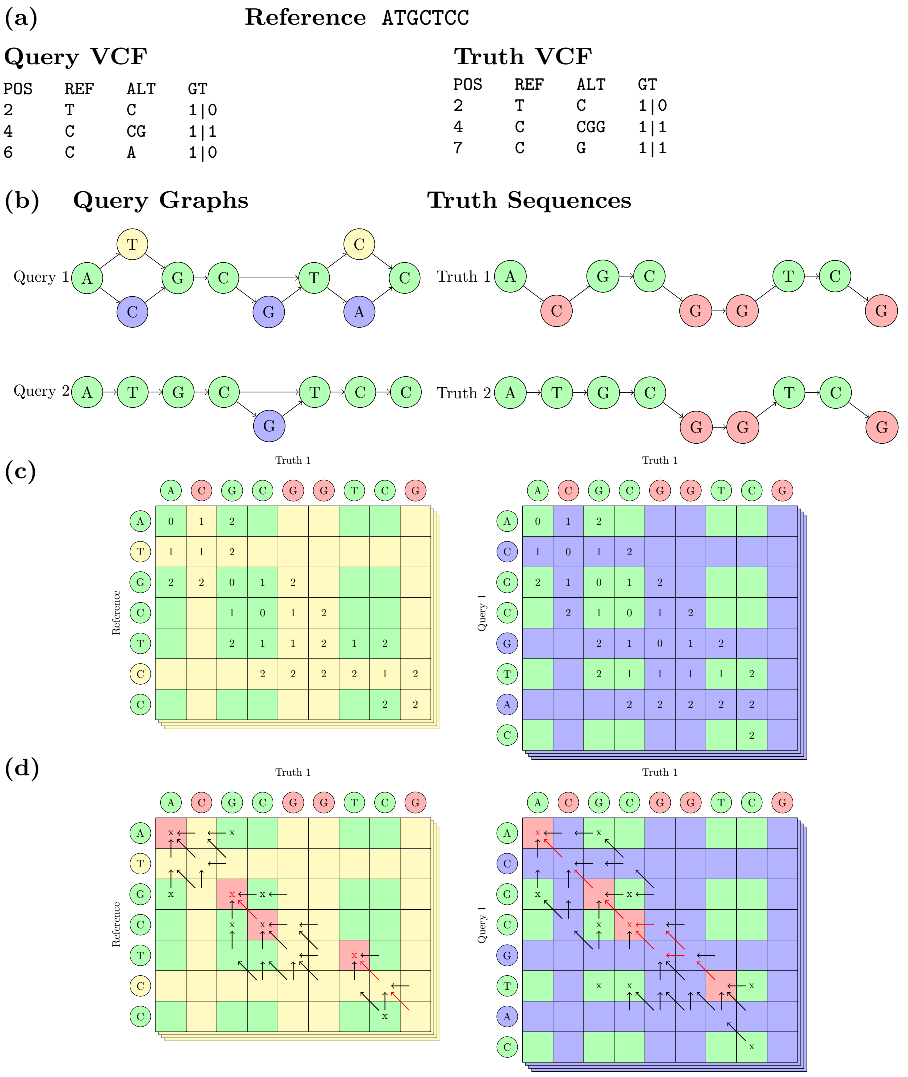

Supplementary Figure 8: **Overview of minimum edit distance algorithm.** False positive query variants are allowed. **(a)** Inputs: reference sequence, phased query VCF, and phased truth VCF **(b)** For truth haplotypes, sequences are generated by applying variants (red) to the reference (green). For query haplotypes, variants (blue) are applied similarly but the reference sequence (green/yellow) is also retained, resulting in a graph. **(c)** For each of the four possible alignments of truth and query haplotypes, the forward-pass Dijkstra minimum edit distance algorithm performs alignment using two matrices. Transitions between the two matrices are allowed when both indices are corresponding reference bases (shown in green). **(d)** During the backwards pass, another Dijkstra search is required to maximize the number of false positive variants, among several possible minimum edit distance paths (red arrows). Corresponding reference bases with a single outgoing path edge are labelled "sync points" (red), and are used for calculating summary statistics.

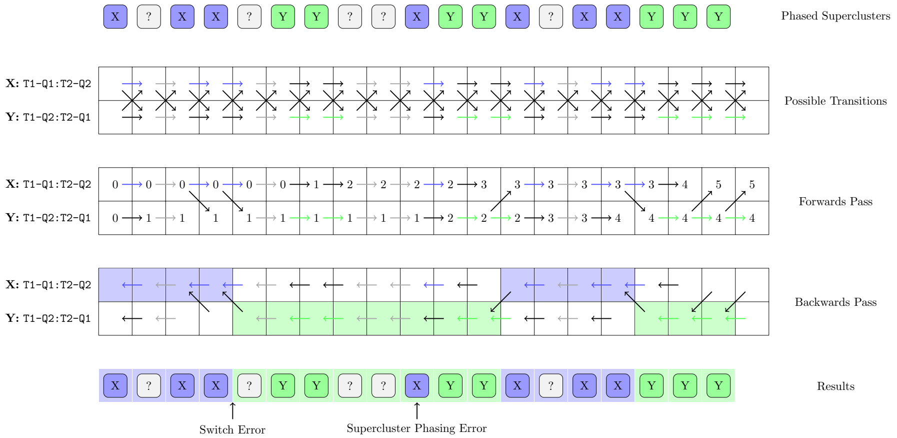

Supplementary Figure 9: **Phasing algorithm overview.** It steps through the two possible relative phasings of truth and query haplotypes (X: Truth1 to Query1 and Truth2 to Query2, or Y: Truth1 to Query2 and Truth2 to Query1) for each supercluster. It minimizes the total number of switch errors and supercluster phasing errors using a dynamic programming algorithm. Black arrows show penalized transitions. Note that either phase state X or Y is allowed in an uncategorized supercluster, and that switch errors are always penalized. Phase blocks are highlighted for phase states X (blue) and Y (green).
