# Supplementary material for: vcfdist: accurately benchmarking phased small variant calls in human genomes
Source: Nat Commun. 2023 Dec 9;14:8149. doi: 10.1038/s41467-023-43876-x (PMC10710436; doi:10.1038/s41467-023-43876-x)
Supplement: Supplementary file 3 — Reporting Summary [file 41467_2023_43876_MOESM3_ESM.pdf]

nature portfolio

Corresponding author(s): Tim Dunn

Last updated by author(s): Nov 1, 2023

Reporting Summary

Nature Portfolio wishes to improve the reproducibility of the work that we publish. This form provides structure for consistency and transparency in reporting. For further information on Nature Portfolio policies, see our Editorial Policies and the Editorial Policy Checklist.

Statistics

For all statistical analyses, confirm that the following items are present in the figure legend, table legend, main text, or Methods section.

n/a	Confirmed	
☒	☐	The exact sample size (*n*) for each experimental group/condition, given as a discrete number and unit of measurement
☒	☐	A statement on whether measurements were taken from distinct samples or whether the same sample was measured repeatedly
☒	☐	The statistical test(s) used AND whether they are one- or two-sided<br>*Only common tests should be described solely by name; describe more complex techniques in the Methods section.*
☒	☐	A description of all covariates tested
☒	☐	A description of any assumptions or corrections, such as tests of normality and adjustment for multiple comparisons
☒	☐	A full description of the statistical parameters including central tendency (e.g. means) or other basic estimates (e.g. regression coefficient) AND variation (e.g. standard deviation) or associated estimates of uncertainty (e.g. confidence intervals)
☒	☐	For null hypothesis testing, the test statistic (e.g. *F*, *t*, *r*) with confidence intervals, effect sizes, degrees of freedom and *P* value noted<br>*Give P values as exact values whenever suitable.*
☒	☐	For Bayesian analysis, information on the choice of priors and Markov chain Monte Carlo settings
☒	☐	For hierarchical and complex designs, identification of the appropriate level for tests and full reporting of outcomes
☒	☐	Estimates of effect sizes (e.g. Cohen's *d*, Pearson's *r*), indicating how they were calculated

Our web collection on statistics for biologists contains articles on many of the points above.

Software and code

Policy information about availability of computer code

Data collection	No software tools were used for data collection.
Data analysis	All code for vcfdist and the benchmarking pipelines developed for this manuscript are available in a public Github repository: https://github.com/TimD1/vcfdist, and deposited in Zenodo under the following DOI: https://zenodo.org/doi/10.5281/zenodo.8368282. Publicly available software used to phase the input variant callsets includes minimap2 v2.24-r1122, samtools v1.16.1, and whatshap v1.7. To evaluate the phased variant callsets, vcfdist v1.0.0, hap.py v0.3.15, and rtg-tools vcfeval v3.12.1 were used.

For manuscripts utilizing custom algorithms or software that are central to the research but not yet described in published literature, software must be made available to editors and reviewers. We strongly encourage code deposition in a community repository (e.g. GitHub). See the Nature Portfolio guidelines for submitting code & software for further information.

Data

Policy information about availability of data

All manuscripts must include a data availability statement. This statement should provide the following information, where applicable:

- Accession codes, unique identifiers, or web links for publicly available datasets
- A description of any restrictions on data availability
- For clinical datasets or third party data, please ensure that the statement adheres to our policy

All datasets used in this manuscript were previously released by other researchers and are publicly available online. The unphased precisionFDA Truth Challenge V2

VCF submissions and corresponding read FASTAs used to phase the VCFs are hosted on the NIST public data repository at https://data.nist.gov/od/id/mds2-2336 (DOI https://doi.org/10.18434/mds2-2336). The phased ground truth VCF and BED files for the HG002 whole-genome and challenging medically-relevant genes datasets are available in the NISTv4.2.1 and CMRG_v1.00 directories of the Genome In A Bottle Consortium's FTP release folder, respectively: https://ftp-trace.ncbi.nlm.nih.gov/ReferenceSamples/giab/release/AshkenazimTrio/HG002_NA24385_son. The GRCh38 reference FASTA is likewise available at https://ftp-trace.ncbi.nlm.nih.gov/ReferenceSamples/giab/release/references/. Source data are provided with this paper.

Research involving human participants, their data, or biological material

Policy information about studies with human participants or human data. See also policy information about sex, gender (identity/presentation), and sexual orientation and race, ethnicity and racism.

Reporting on sex and gender	This manuscript did not report on sex and gender.
Reporting on race, ethnicity, or other socially relevant groupings	This manuscript did not report on race, ethnicity, or other groupings.
Population characteristics	This study did not collect data from human research participants.
Recruitment	This study did not recruit participants.
Ethics oversight	This study did not require approval, as it only contains data analyses.

Note that full information on the approval of the study protocol must also be provided in the manuscript.

Field-specific reporting

Please select the one below that is the best fit for your research. If you are not sure, read the appropriate sections before making your selection.

☒ Life sciences ☐ Behavioural & social sciences ☐ Ecological, evolutionary & environmental sciences

For a reference copy of the document with all sections, see nature.com/documents/nr-reporting-summary-flat.pdf

Life sciences study design

All studies must disclose on these points even when the disclosure is negative.

Sample size	For this manuscript, whole genome sequencing results for a single human genome (HG002) using 64 different variant calling pipelines were compared. We used all 64 VCFs provided in a public data repository by the PrecisionFDA following their variant calling challenge "Truth Challenge V2: Calling Variants from Short and Long Reads in Difficult-to-Map Regions", hosted in 2020. This dataset represents a variety of whole genome sequencing technologies and pipelines for evaluating our benchmarking software.
Data exclusions	No data were excluded. Several VCFs required modifying metadata in order to run, due to an incorrect initial formatting.
Replication	In the Github repository (https://github.com/TimD1/vcfdist), BASH scripts are provided which can be used to re-run any stage of the pipeline used for processing data in this study. Analyses have been successfully reproduced by the authors.
Randomization	No randomization was involved in this study because all VCFs were evaluated using both our method (vcfdist) and the baseline (vcfeval).
Blinding	No blinding was involved in this study because all VCFs were evaluated using both out method (vcfdist) and the baseline (vcfeval).

Reporting for specific materials, systems and methods

We require information from authors about some types of materials, experimental systems and methods used in many studies. Here, indicate whether each material, system or method listed is relevant to your study. If you are not sure if a list item applies to your research, read the appropriate section before selecting a response.

Materials & experimental systems

n/a	Involved in the study
☒	☐ Antibodies
☒	☐ Eukaryotic cell lines
☒	☐ Palaeontology and archaeology
☒	☐ Animals and other organisms
☒	☐ Clinical data
☒	☐ Dual use research of concern
☒	☐ Plants

Methods

n/a	Involved in the study
☒	☐ ChIP-seq
☒	☐ Flow cytometry
☒	☐ MRI-based neuroimaging
